# Supplementary material for: Antigen-selective modulation of AAV immunogenicity with tolerogenic rapamycin nanoparticles enables successful vector re-administration
Source: Nat Commun. 2018 Oct 5;9:4098. doi: 10.1038/s41467-018-06621-3 (PMC6173722; doi:10.1038/s41467-018-06621-3)
Supplement: Supplementary file 1 — Supplementary Information [file 41467_2018_6621_MOESM1_ESM.pdf]

Antigen-selective modulation of AAV immunogenicity with tolerogenic rapamycin nanoparticles enables successful vector re-administration

Meliani et al. Supplementary Information:

Supplementary Figure 1-11 Page 2

Supplementary Tables 1-4 Page 15

Supplementary Figures

Supplementary Figure 1

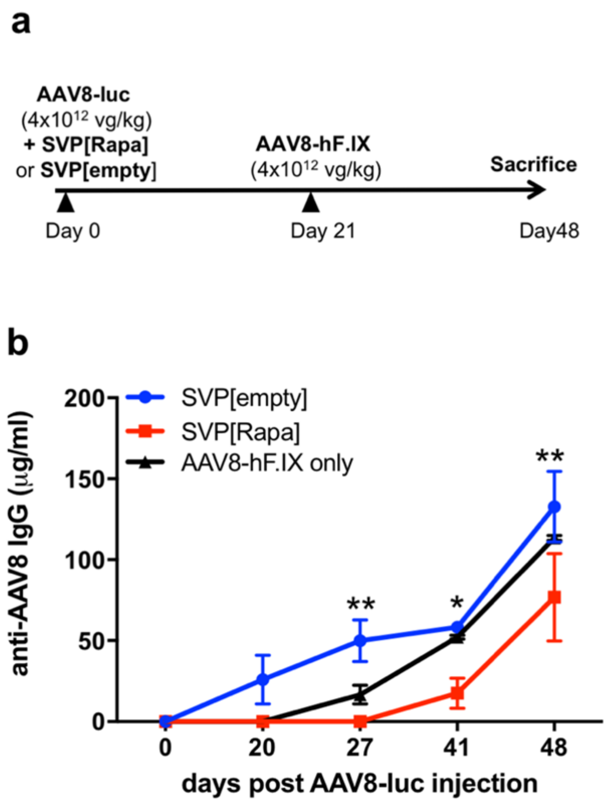

Supplementary Figure 1 SVP[Rapa] treatment is required to fully control anti-AAV antibody formation. (**a**) Protocol outline. Male C57BL/6 mice (n=5) were first injected i.v. with $4x10^{12}$vg kg^{-1} of AAV8-luc vector together with SVP[Rapa] (200 µg) or with SVP[empty] control. Three weeks later, animals were then challenged i.v. with $4x10^{12}$ vg kg^{-1} of AAV8-hF.IX vector only. (**b**) Kinetic analysis of anti-AAV8 IgG antibodies measured by ELISA. Data are shown as mean ± s.e.m. (* p <0.05, ** p <0.01, two-way ANOVA with Tukey's multiple comparison test). SVP[Rapa] treatment consisted of 200 µg of rapamycin.

Supplementary Figure 2

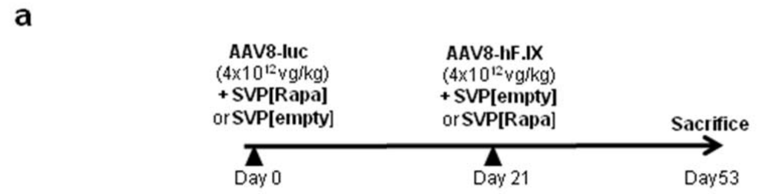

Cohort	Group	Injection on day 0	Re-injection on day 21
	SVP[Rapa]/SVP[empty]	AAV8-luc (4x10¹² vg/kg) +SVP[Rapa]	AAV8-hF.IX (4x10¹² vg/kg) + SVP[empty]
	SVP[empty]/SVP[Rapa]	AAV8-luc (4x10¹² vg/kg) +SVP[empty]	AAV8-hF.IX(4x10¹² vg/kg) + SVP[Rapa]

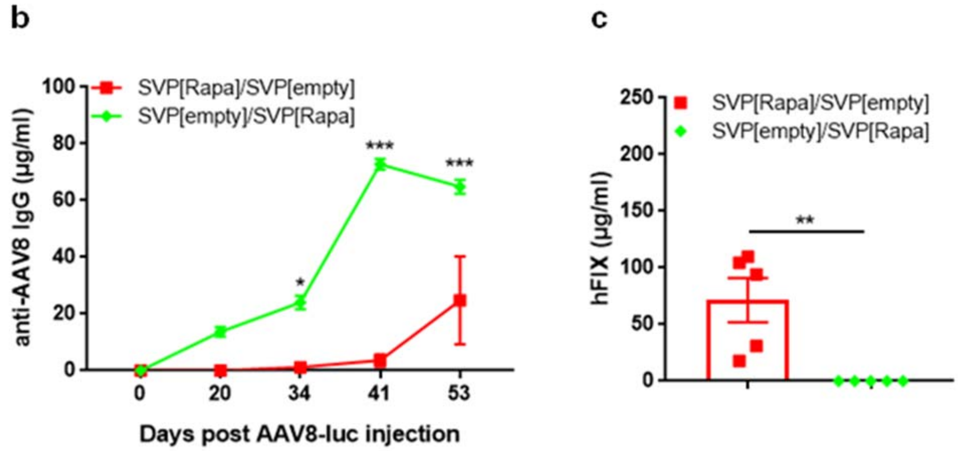

Supplementary Figure 2 **SVP[Rapa] treatment is required to fully control anti-AAV antibody formation. a** protocol outline. Male C57BL/6 (n=5) were treated intravenously (iv) with an AAV8-Luc vector ($4x10^{12}$ vg/kg) together with SVP[Rapa] or SVP[empty] control. Three weeks later, animals received an AAV8-hF.IX vector ($4x10^{12}$ vg/kg) together with SVP[Rapa] or SVP[empty] control. **b** Anti-AAV8 IgG antibodies measured by ELISA. Data are shown as mean ± SEM (n=5, *, p<0.05, ***, p<0.001, one-way ANOVA with Bonferroni post-test). **c** hF.IX antigen levels in plasma at day 54 determined by ELISA. The symbols represent individual animals and bars represent mean ± SEM (n=5, **, p<0.01, two-tailed unpaired Mann-Whitney U test). SVP[Rapa] treatment consisted of 200 µg of rapamycin.

Supplementary Figure 3

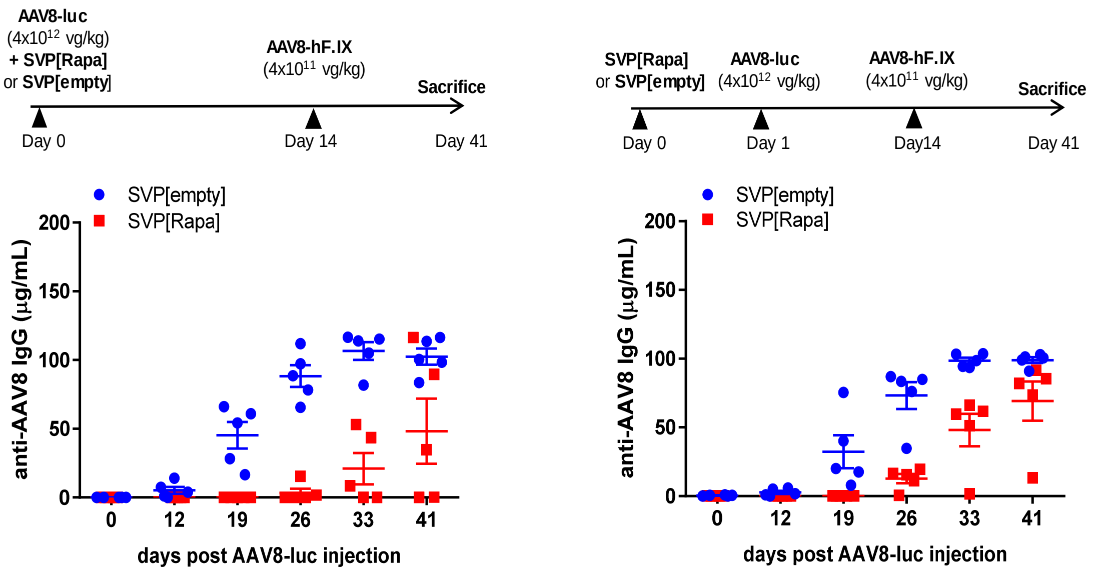

Supplementary Figure 3 Role of timing of SVP[Rapa] administration in the control of anti-AAV antibody formation in mice. Upper graphs, study design. In one study (left), mice received an AAV8-luc vector with SVP[Rapa] or SVP[empty] control at day 0. After 2 weeks animals were challenged with an AAV8-hF.IX vector. In a second study, mice received SVP[Rapa] or SVP[empty] control at day 0 and an AAV8-luc vector at day 1. After 2 weeks animals were challenged with an AAV8-hF.IX vector. Lower panels show that animals treated with SVP[Rapa] one day before AAV8-luc administration had higher titer anti-AAV8 antibodies after challenge than animals that received SVP[Rapa] together with the AAV8-luc vector at day 0.

Supplementary Figure 4

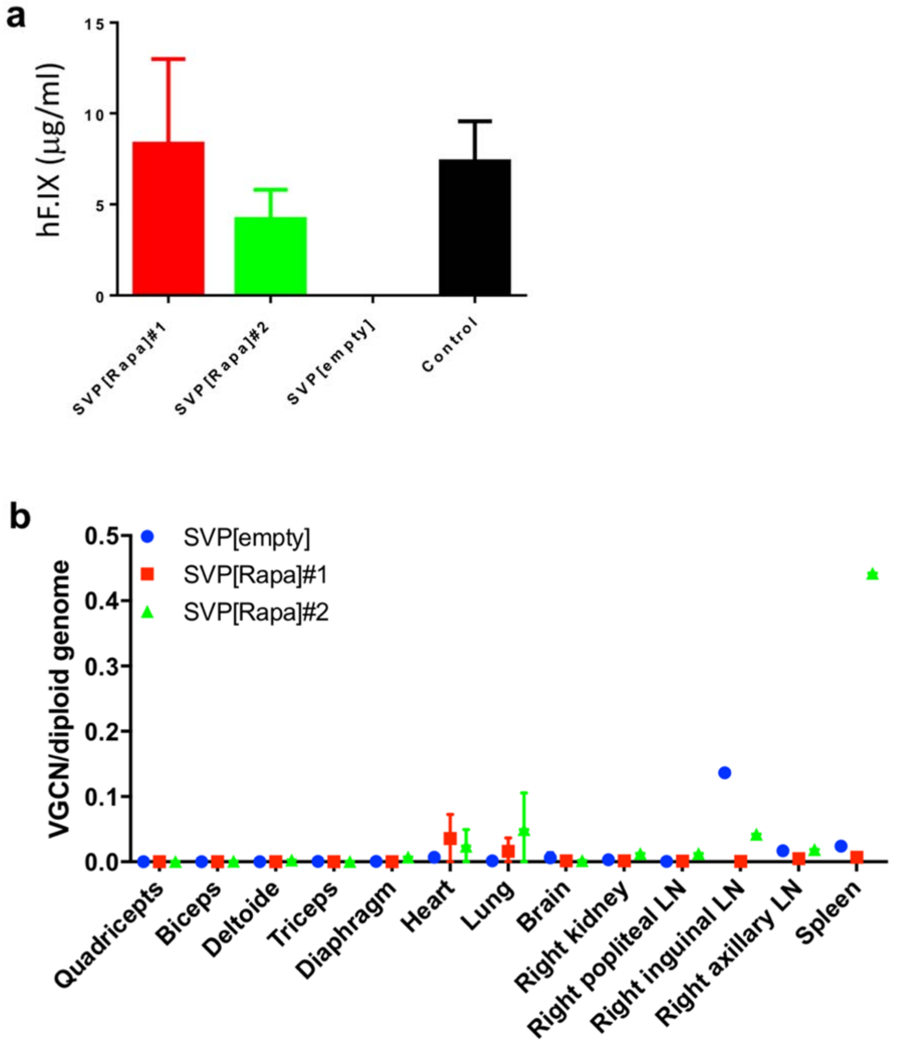

Supplementary Figure 4 SVP[Rapa] treatment in nonhuman primates (NHPs). (**a**) Neutralizing activity of sera from AAV-treated NHPs injected in mice. Sera from NHP described in Figure 2 were collected 30 days post administration of AAV8-Gaa vector ($2x10^{12}$ vg kg^{-1}) and SVP[Rapa] (3mg kg^{-1} of rapamycin, n=2, SVP[Rapa]#1 and SVP[Rapa]#2) or SVP[empty] control (n=1). Serum from each NHP was then injected into C57BL/6 mice (n=5 per group). As control, an additional group of mice was left untreated (n=5). All animals were then injected one day later with AAV8-hF.IX vector ($4x10^{11}$ vg kg^{-1}). h.FIX levels measured 14 days post AAV8-hF.IX injection. Data are shown as mean ± s.e.m.. (**b**) Biodistribution of AAV in NHP is not affected by SVP[Rapa] treatment. NHP tissues were collected at sacrifice (day 95 post infusion with AAV8-Gaa vector with SVP[Rapa] or with SVP[empty] control) to perform biodistribution. Results expressed as vector genome copy number (VGCN) per diploid genome (mean ± s.e.m. of testing replicates).

Supplementary Figure 5

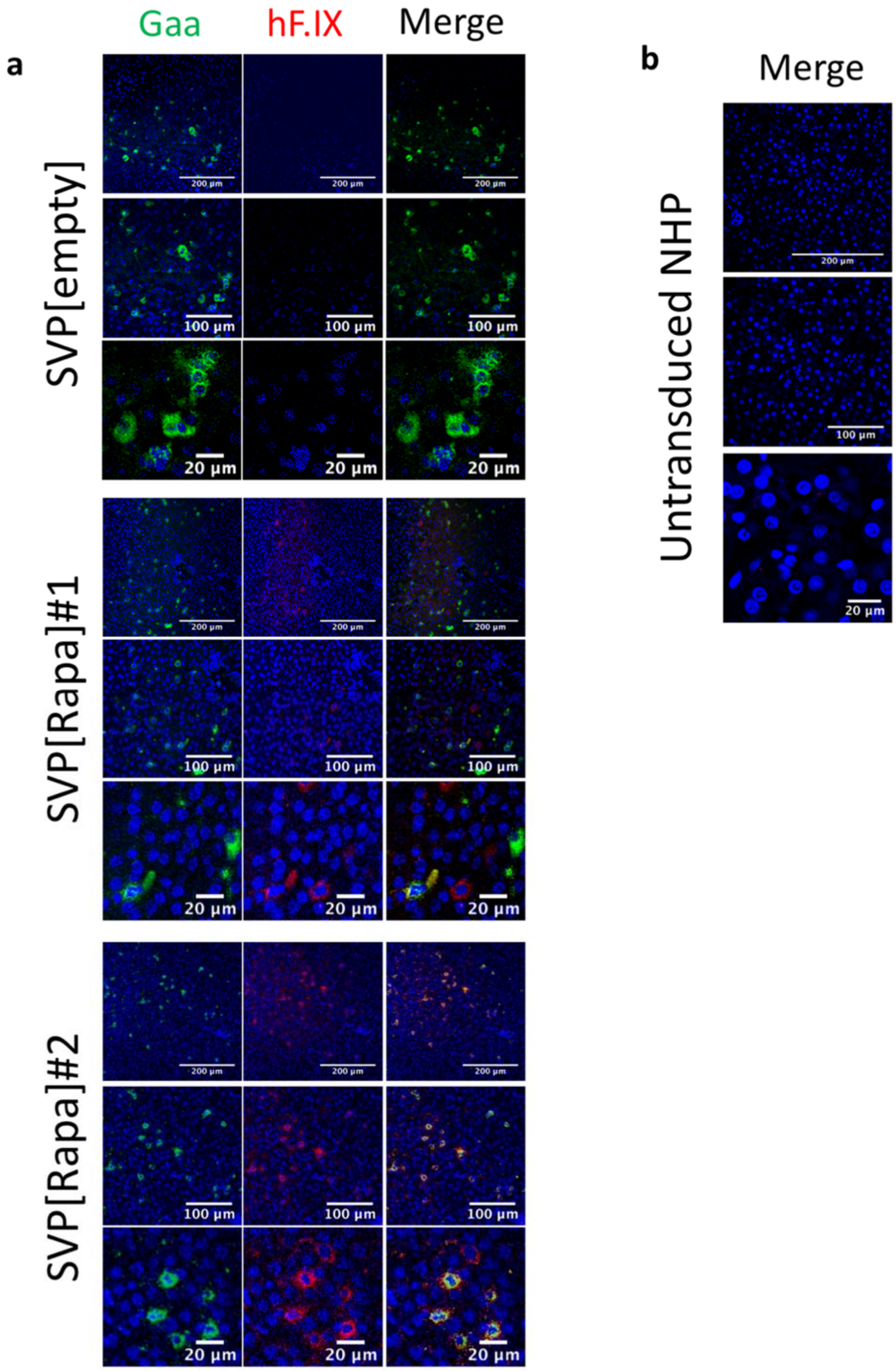

Supplementary Figure 5 Enhanced liver transduction via repeated AAV8 vector administrations in nonhuman primates. (**a**) Dual immunofluorescence staining of Gaa and hF.IX in livers from nonhuman primates treated with AAV8 vectors together with SVP[Rapa]

(n=2, SVP[Rapa]#1 and SVP[Rapa]#2, 3 mg kg^{-1}) or SVP[empty] control (n=1). 95 days post AAV8-Gaa administration, liver lobes were collected and stained for Gaa (shown in green), hF.IX (shown in red) and Gaa/hF.IX co-localization (merge, shown in yellow) and nuclei are DAPI-stained (Blue). Shown are images of individual staining and merged images for colocalization. Scale bars correspond to 200, 100, and 20 µm. (**b**) Merge images of liver costained for Gaa and hF.IX in a non-injected monkey. Scale bars correspond to 200, 100, and 20 µm. Nuclei are DAPI-stained (Blue).

Supplementary Figure 6

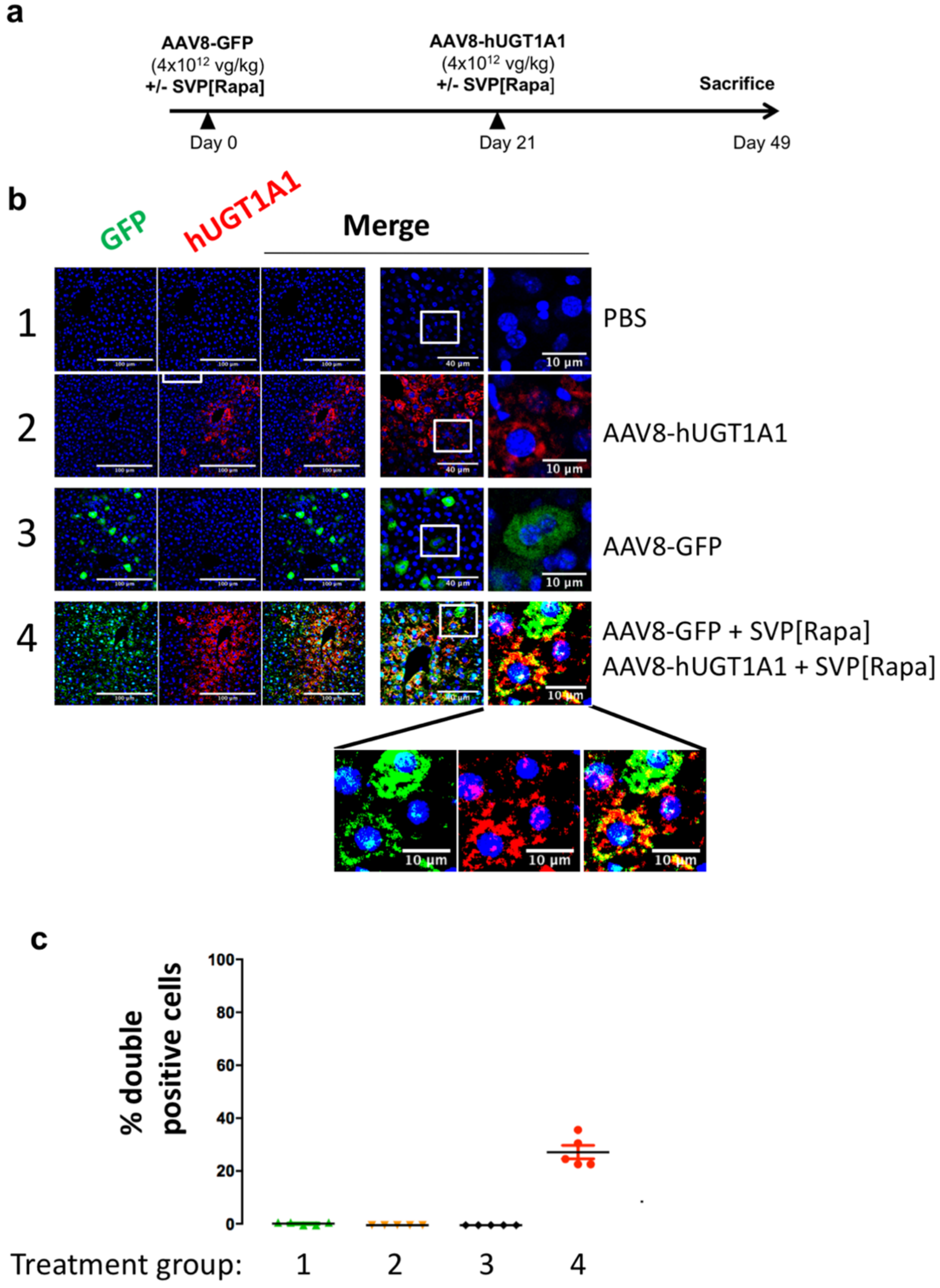

Supplementary Figure 6 Enhanced liver transduction via repeated AAV8 vector administrations in mice. (**a**) Protocol outline**.** Male C57BL/6 mice (n=5) were treated i.v. with $4x10^{12}$ vg kg^{-1} of AAV8-GFP vector alone (control) or together with SVP[Rapa] on day 0. Three weeks later, animals were challenged i.v with $4x10^{12}$ vg kg^{-1} of AAV8-hUTG1A1 vector alone (control) or with SVP[Rapa] (200 µg). As a control for GFP expression, an additional group (n=5) was injected iv with $4x10^{12}$ vg kg^{-1} of AAV8-GFP vector alone on day 0 and three weeks later with PBS (group AAV8-GFP). Similarly, for hUGT1A1, mice (n=5) were injected on day 0 with PBS and three weeks later with $4x10^{12}$ vg kg^{-1} of AAV8-UGT1A1 vector alone (group AAV8-hUGT1A1). A group of mice injected with PBS only served as staining control. (**b**) Representative images of immunostaining of hUGT1A1 and GFP performed in livers collected 49 days post treatment. GFP is shown in green, hUGT1A1 is shown in red, GFP/UGT1A1 co-localization (merge) in yellow, and nuclei are DAPI-stained (blue). Scale bars correspond to 100 and 10 µm. Treatment groups are indicated, Group 1, PBS control; Groups 2, AAV8-hUGT1A1 vector only; Groups 3, AAV8-GFP vector only; Groups 2, AAV8-GFP/AAV8-hUGT1A1 vectors. (**c**) quantification of the % of double positive (GFP^+ and $UGT1A1^+$ hepatocytes in the different treatment groups. Data are shown as mean ± s.e.m.

Supplementary Figure 7

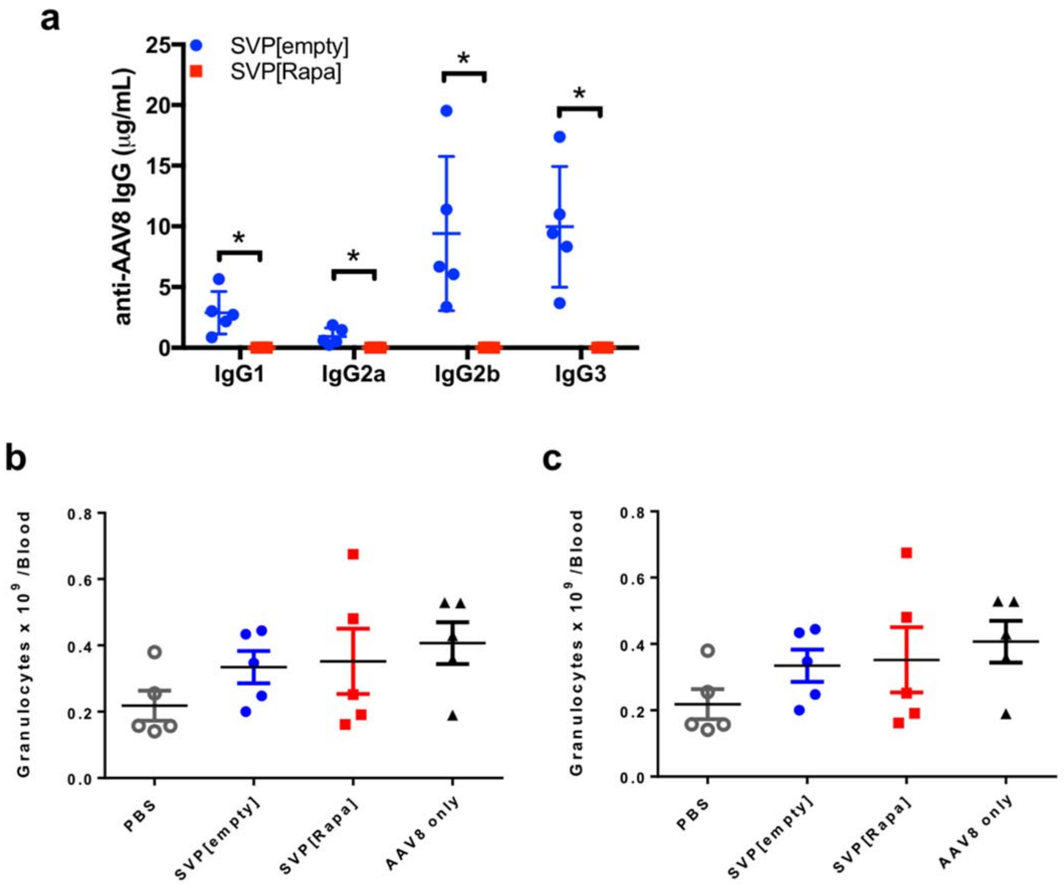

Supplementary Figure 7 Efficacy and safety of SVP[Rapa] co-administration with AAV8 vector *in vivo*. (**a-c**) Male C57BL/6 mice (n=5, from Figure 4b-d) were treated with $4x10^{12}$ vg kg^{-1} of AAV8 vector encoding the VP1 structural protein of the AAV8 capsid (AAV8-VP1) together with SVP[Rapa] (200 µg) or with SVP[empty] control. (**a**) Analysis of anti-AAV8 IgG subclasses performed 14 days post vector injection and measured by ELISA. (**b**) Frequency of monocytes and (**c**) of granulocytes determined in peripheral blood 14 days post vector injection using an MS9 automated cell counter. All data are shown as mean ± s.e.m of n=5 animals per group. SVP[Rapa] treatment consisted of 200 µg of rapamycin. * *p* <0.05; two-way ANOVA with Tukey's multiple comparison test.

Supplementary Figure 8

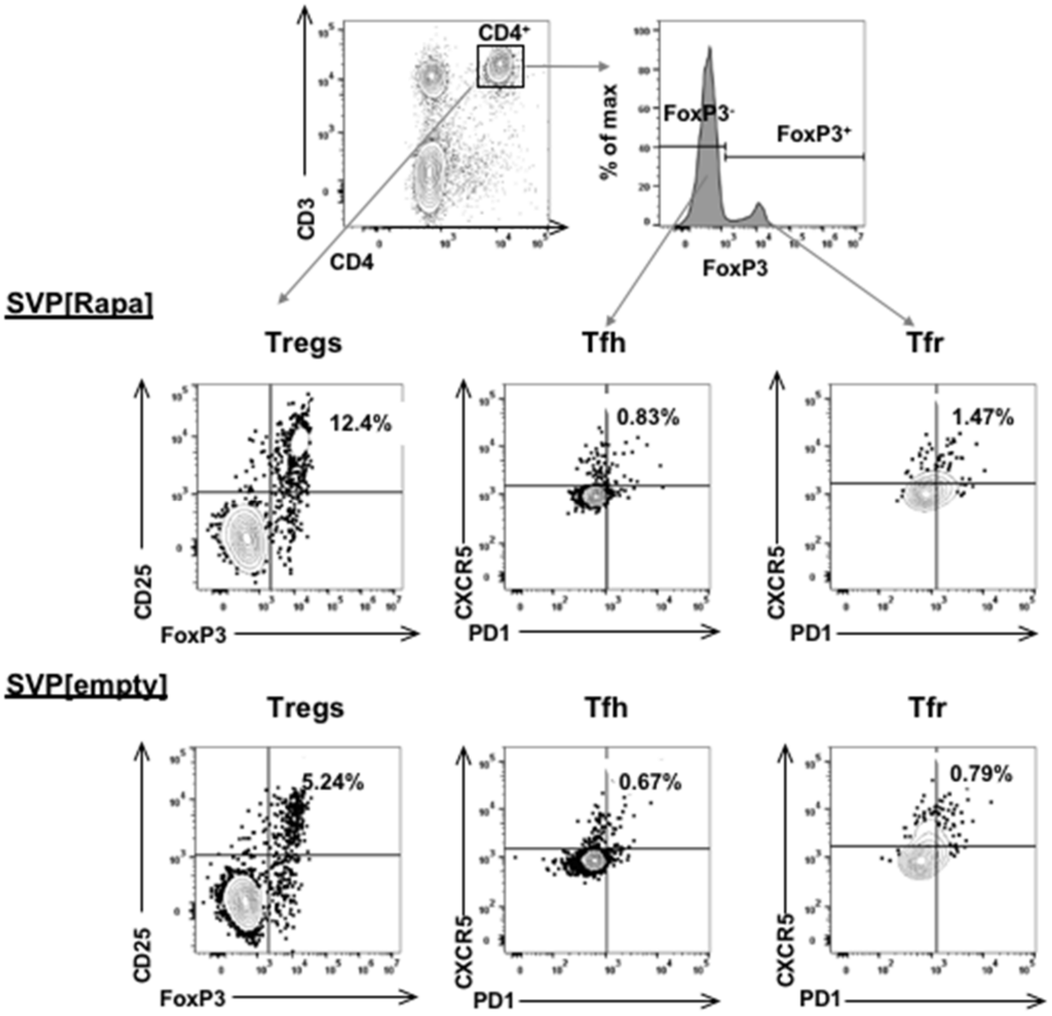

Supplementary Figure 8 Flow cytometry gating strategy for the identification of Tregs and CXCR5$^+$ follicular T cells. Male C57BL/6 mice (n=5) were treated with $4x10^{12}$ vg kg^{-1} of AAV8-luc vector together with SVP[Rapa] (200 µg) or with SVP[empty] control. Representative flow cytometry gating strategy for the identification of Tregs defined as CD4$^+$ CD25$^+$ FoxP3$^+$, Tfr defined as CD4$^+$ FoxP3$^+$ CXCR5$^+$ PD-1$^+$ and Tfh defined as CD4$^+$ FoxP3$^-$ CXCR5$^+$ PD-1$^+$. Isotype control antibodies were used to set the gates for FoxP3, CXCR5 and PD-1.

Supplementary Figure 9

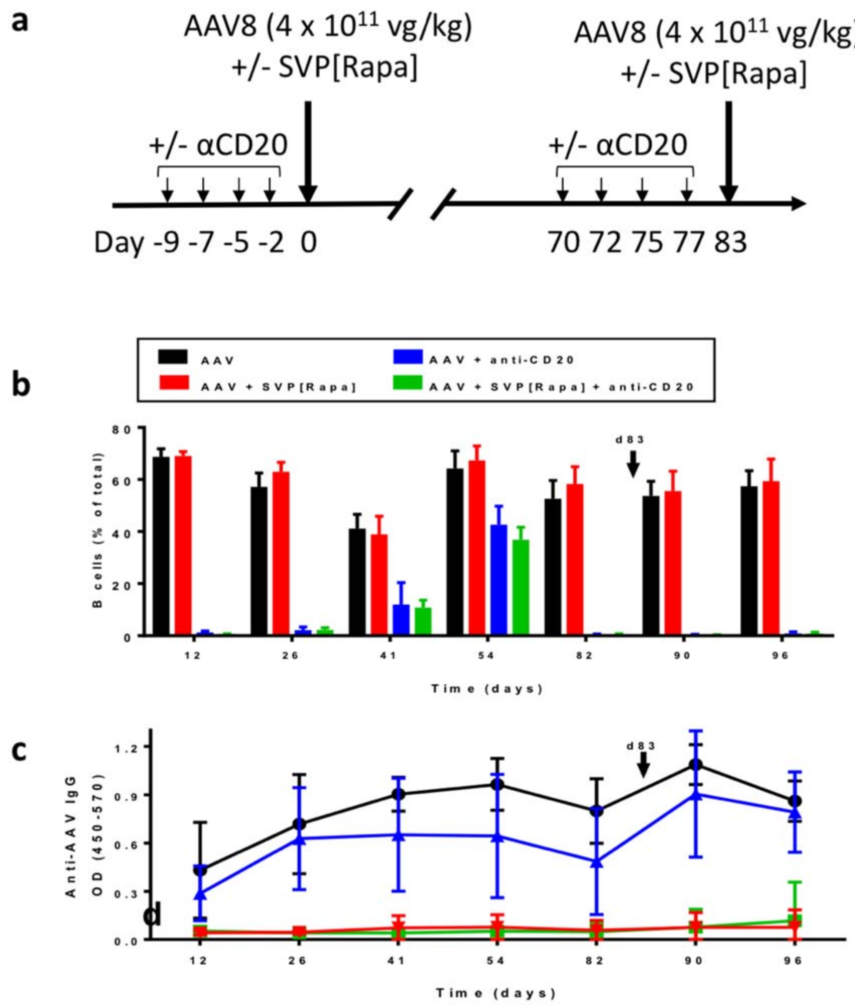

Supplementary Figure 9 SVP[Rapa] but not B-cell depletion prevents anti-AAV8 antibody formation. (**a**) Protocol outline. Female C57BL/6 mice (n=6) were treated i.v. with an AAV8 vector (4 x 10^{11} vg kg^{-1}) and then 83 days later with the same dose of AAV8 vector. One group of mice received no additional treatment (Black bars and lines), a second group received SVP[Rapa] (50 μg) with each dose of AAV8 (Red bars and lines), a third group received 4 injections of a depleting mouse anti-mouse CD20 antibody prior to each injection of AAV8 (Blue bars and lines), and a fourth group received both SVP[Rapa] and anti-CD20 antibody (Green bars and lines). SVP[Rapa] treatments at days 0 and 83 consisted of 50 μg of rapamycin. Anti-CD20 antibody (αCD20, 250 μg/mouse, i.v.) was administered on days -9, -7, -5 and -2 days and days 70, 72, 75, and 83. (**b**) B cell frequency over time after anti-CD20 and SVP[Rapa] treatment. Whole blood was collected at times shown and analyzed by flow

cytometry for CD19$^+$ B cells. (**c**) Anti-AAV8 IgG over time measured by ELISA. The second injection of AAV8 on day 83 is indicated with an arrow. All data are shown as mean ± s.e.m.

Supplementary Figure 10

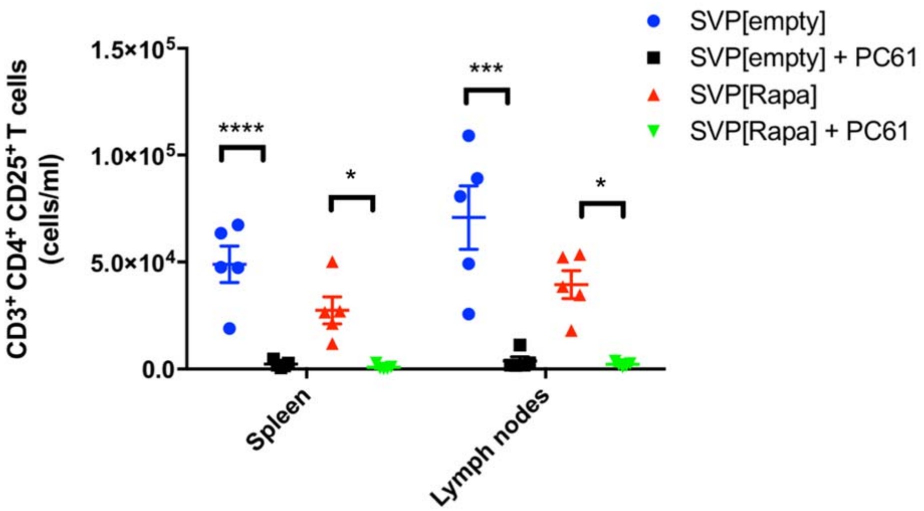

Supplementary Figure 10 Efficient depletion of Tregs using anti-CD25 antibody. Male C57BL/6 mice (n=5) were treated as described in Fig 6g,h. The AAV8-Luc vector (4x10^{12} vg kg^{-1}) together with SVP[Rapa] (200 µg) or SVP[empty] control was administered on day 0. Tregs were depleted with anti-CD25 antibody (clone PC61) treatment on days 19 and 20 prior to administration of AAV8-hF.IX (4x10^{12} vg kg^{-1}) together with SVP[Rapa] or SVP[empty] control on day 21. CD3$^+$ CD4$^+$ CD25$^+$ T cell count in spleen and inguinal lymph nodes was performed at the sacrifice on day 32 post AAV8-luc vector injection. Data are plotted as individual animals and bars represent mean ± s.e.m. SVP[Rapa] treatment consisted of 200 µg of rapamycin.

Supplementary Figure 11

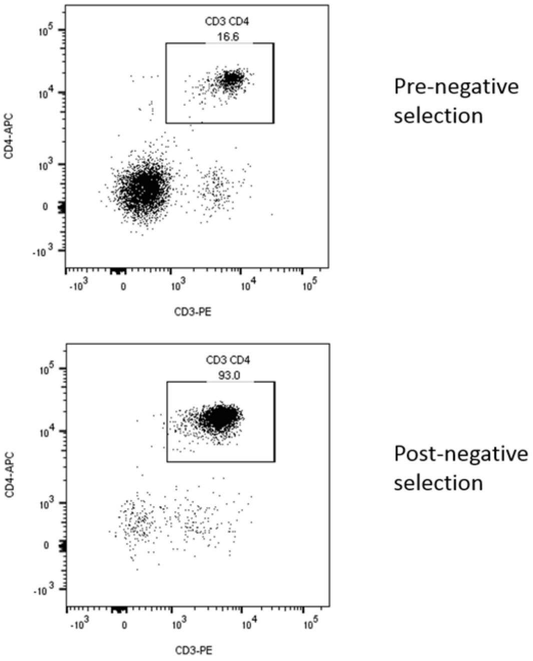

Supplementary Figure 11 Flow cytometry plot showing the negative selection of CD4+ T cells prior to transfer into recipient mice. Upper plot, splenocytes stained for CD4-APC and CD3-PE prior to negative selection of $CD4^+$ T cells. Lower plot, staining after selection. The square in the graphs show the % of $CD3^+CD4^+$ T cells.

Supplementary Table 1. Clinical chemistry and hematology parameters SVP[Rapa] #1.

SVP[Rapa] #1		Normal values	D-12	D3	D15	D31	D45	D57	D73	D95
Urea	g/L	[0.2-0.6]	0.3	0.3	0.3	0.3	0.3	0.3	0.3	0.2
Creatinin	mg/L	[4-9]	8	8	8	7	10	7	7	8
Alkaline phosphatase	U/L	<2000	1944	1827	1442	1027	1156	1275	1402	>2000
ALT or SGPT	U/L	[16-120]	41	40	46	40	28	42	39	39
AST or SGOT	U/L	[15-70]	39	38	41	33	40	33	33	35
Albumin	g/L	[34-47]	41	42	39	40	38	34	37	40
Total proteins	g/L	[65-85]	70	75	78	67	81	68	65	66
Globulin	g/L	-	29	33	39	27	43	34	28	26
Albumin / globulin		>0.8	1.4	1.3	1.0	1.5	0.9	1.0	1.3	1.5
Gamma GT	U/L	<220	360	298	253	290	164	197	267	371
Glucose	g/L	[0.6-1.3]	0.5	0.8	0.5	0.5	0.5	0.7	0.6	0.8
Total cholesterol	g/L	[0.7-2.0]	0.7	0.8	1.0	0.8	0.7	0.8	0.7	0.7
Triglycerides	g/L	-	0.5	0.6	1.1	0.3	1.7	0.5	0.6	0.5
Total bilirubin	mg/L	[1-4]	<1	2	0.7	1	3	1	3	<1
Calcium	mg/L	[90-115]	109	107	109	100	112	107	106	100
Inorganic phosphorus	mg/L	[34-76]	52	61	46	48	52	48	51	45
LDH	U/L	-	753	621	787	663	727	692	736	577
Sodium	mmol/L	[143-163]	155	153	160	156	155	158	156	157
Potassium	mmol/L	[3.8-6.0]	3.5	3.2	3.4	2.8	3.3	3.1	3.2	3.5
Chloride	mmol/L	[101-121]	111	111	113	111	110	112	111	113
C reactive protein	ng/ml	<2000	495	2270	895	870	7900	1220	1385	385
Red blood cells	106/L	[4.5-6.5]	6.8	6.9	6.4	5.9	5.5	5.5	6.0	6.8
Hemoglobin	g/L	[108-150]	135	137	124	113	100	99	110	125
Hematocrit	%	[36-48]	44	44	42	38	35	34	39	44
MCV	fl	[58-71]	65.8	65.4	65.4	64.6	63.6	61.5	63.9	64.3
MCH	pg	[19.5-27.0]	19.9	19.9	19.3	19.1	18.2	18.0	18.2	18.5
MCHC	%	[28.0-33.0]	30.7	31.1	29.5	29.7	28.7	29.2	28.5	28.7
Reticulocyte	10^3/L	<80	38.0	15.9	12.9	121.7	19.2	154.3	119.0	51.4
Leucocytes	10^3/L	[5.7-21.0]	11.1	20.4	10.3	15.0	17.7	22.8	12.0	19.7
Neutrophils	10^3/L	[3.5-8.5]	2.7	13.1	3.3	4.3	8.8	12.8	4.0	11.4
Lymphocytes	10^3/L	[3.4-7.8]	7.9	5.9	6.1	10.2	7.6	8.6	7.1	7.3
Monocytes	10^3/L	[0.4-1.3]	0.1	1	0.7	0.5	1.1	1.1	0.6	1.0
Eosinophils	10^3/L	<0.7	0.3	0.4	0.2	0	0.2	0.3	0.3	0
Basophils	10^3/L	<0.1	0.1	0	0	0	0	0	0	0
Platelets	10^3/L	[270-580]	347	347	515	366	492	157	347	375

Supplementary Table 2. Clinical chemistry and hematology parameters SVP[Rapa] #2.

SVP[Rapa] #2		Normal values	D-12	D3	D15	D31	D45	D57	D73	D95
Urea	g/L	[0.2-0.6]	0.4	0.3	0.4	0.4	0.4	0.3	0.3	0.3
Creatinin	mg/L	[4-9]	7	8	7	7	8	8	8	8
Alkaline phosphatase	U/L	<2000	1452	1382	1139	1159	1186	1328	1708	1176
ALT or SGPT	U/L	[16-120]	55	52	57	69	51	52	51	63
AST or SGOT	U/L	[15-70]	35	40	52	52	48	53	36	51
Albumin	g/L	[34-47]	38	37	36	38	35	35	34	33
Total proteins	g/L	[65-85]	70	78	76	70	73	69	68	64
Globulin	g/L	-	32	41	40	32	38	34	34	31
Albumin / globulin		>0.8	1.2	0.9	0.9	1.2	0.9	1.0	1.0	1.1
Gamma GT	U/L	<220	214	149	148	187	143	134	132	144
Glucose	g/L	[0.6-1.3]	0.6	0.5	0.6	0.5	0.6	0.5	0.2	0.8
Total cholesterol	g/L	[0.7-2.0]	1.1	1.5	1.4	1.4	1.3	1.3	1.2	1.1
Triglycerides	g/L	-	0.9	0.6	1.3	0.4	0.9	0.9	1.2	0.9
Total bilirubin	mg/L	[1-4]	1	2	1	1	1	< 1	1	< 1
Calcium	mg/L	[90-115]	103	107	104	100	102	105	108	95
Inorganic phosphorus	mg/L	[34-76]	55	43	35	45	41	40	43	45
LDH	U/L	-	945	790	1066	1098	984	1142	1084	1031
Sodium	mmol/L	[143-163]	155	155	158	159	153	158	153	157
Potassium	mmol/L	[3.8-6.0]	3.2	3.1	3.1	3.2	2.8	3.3	3.1	3.6
Chloride	mmol/L	[101-121]	112	110	114	115	112	115	109	115
C reactive protein	ng/ml	<2000	75	4820	635	290	905	105	145	135
Red blood cells	10^6/L	[4.5-6.5]	6.7	7.2	6.6	6.1	6.4	7.0	6.9	6.3
Hemoglobin	g/L	[108-150]	123	130	118	105	108	117	113	105
Hematocrit	%	[36-48]	41	45	38	37	38	41	40	37
MCV	fl	[58-71]	63.3	62.1	60.2	59.6	60	58.4	58.8	59.5
MCH	pg	[19.5-27.0]	18.5	18.1	17.9	17.2	16.8	16.6	16.5	16.8
MCHC	%	[28.0-33.0]	30	28.9	31.1	28.4	28.4	28.5	28	28.2
Reticulocyte	10^3/L	<80	35.9	2.9	19.1	76.4	31.5	23.9	54.1	17.5
Leucocytes	10^3/L	[5.7-21.0]	9.7	8.8	7.4	9.6	11	11.2	12.4	13.2
Neutrophils	10^3/L	[3.5-8.5]	1.3	4.1	2.6	3.3	7	3.1	3.5	6.9
Lymphocytes	10^3/L	[3.4-7.8]	7.4	4.1	3.8	6.1	3.5	6.8	8.4	5.7
Monocytes	10^3/L	[0.4-1.3]	0.6	0.6	0.7	0.1	0.3	1.1	0.5	0.5
Eosinophils	10^3/L	<0.7	0.3	0	0.3	0.1	0.2	0.2	0	0.1
Basophils	10^3/L	<0.1	0.1	0	0	0	0	0	0	0
Platelets	10^3/L	[270-580]	228	176	182	193	273	139	240	247

Supplementary Table 3. Clinical chemistry and hematology parameters SVP[empty].

SVP[empty]		Normal values	D-12	D3	D15	D30	D45	D60	D70	D90
Urea	g/l	[0.2-0.6]	0.3	0.3	0.3	0.4	0.3	0.3	0.3	0.2
Creatinin	mg/l	[4-9]	7	7	6	7	8	7	8	8
Alkaline phosphatase	U/l	<2000	>2000	>2000	>2000	>2000	>2000	>2000	>2000	>2000
ALT or SGPT	U/l	[16-120]	76	56	55	76	69	55	59	61
AST or SGOT	U/l	[15-70]	45	40	41	61	49	36	47	42
Albumin	g/l	[34-47]	43	41	41	43	43	41	40	36
Total proteins	g/l	[65-85]	68	65	66	67	70	67	62	57
Globulin	g/l	-	25	24	25	24	27	26	22	21
Albumin / globulin		>0.8	1.7	1.7	1.6	1.8	1.6	1.6	1.8	1.7
Gamma GT	U/l	<220	341	226	223	243	226	233	239	237
Glucose	g/l	[0.6-1.3]	0.7	0.7	0.7	0.5	0.6	0.6	0.7	1.0
Total cholesterol	g/l	[0.7-2.0]	0.9	0.9	0.9	0.9	1.0	0.9	0.8	0.8
Triglycerides	g/l	-	0.6	0.4	0.6	0.3	0.5	0.5	0.5	0.5
Total bilirubin	mg/l	[1-4]	1	1	1.4	1	<1	1	1	1
Calcium	mg/l	[90-115]	110	100	100	96	105	103	102	95
Inorganic phosphorus	mg/l	[34-76]	49	51	43	76	48	51	48	58
LDH	U/l	-	838	587	646	828	812	713	827	641
Sodium	mmol/l	[143-163]	158	156	159	160	156	158	157	156
Potassium	mmol/l	[3.8-6.0]	4.0	3.3	3.3	4.1	4.1	3.6	3.0	3.9
Chloride	mmol/l	[101-121]	114	113	117	118	115	115	114	113
C relative protein	ng/ml	<2000	180	0	165	350	15	95	155	5
Red blood cells	10^6/µl	[4.5-6.5]	6.3	6.1	5.6	6.0	6.0	5.8	5.7	5.6
Hemoglobin	g/l	[108-150]	127	122	113	122	122	118	116	113
Hematocrit	%	[36-48]	43	39	37	39	40	40	40	38
MCV	fl	[58-71]	67.3	67.1	67.7	68.9	70.1	69.1	69.6	68.4
MCH	pg	[19.5-27.0]	20.1	20.2	20.3	20.2	20.5	20.5	20.3	20.3
MCHC	%	[28.0-33.0]	29.5	31.3	30.5	31.3	30.5	29.6	29.1	29.7
Reticulocyte	10^3/µl	<80	46.8	36.3	48.5	62.8	51.8	53.0	66.4	50.1
Leucocytes	10^3/µl	[5.7-21.0]	9.6	9.9	10.4	13.0	9.4	8.0	9.6	7.4
Neutrophils	10^3/µl	[3.5-8.5]	3.4	5.4	4.2	8.0	1.7	1.8	2.0	2.9
Lymphocytes	10^3/µl	[3.4-7.8]	6.0	4.0	5.5	4.9	6.9	5.8	6.7	4.0
Monocytes	10^3/µl	[0.4-1.3]	0	0.3	0.4	0	0.7	0.2	0.6	0.4
Eosinophils	10^3/µl	<0.7	0.1	0.2	0.3	0	0.1	0.1	0.3	0.1
Basophils	10^3/µl	<0.1	0.1	0	0	0.1	0	0.1	0	0
Platelets	10^3/µl	[270-580]	231	307	375	208	153	246	294	414

Supplementary Table 4. Primers and probes used in the study

hAAT	Forward	5'-GGCGGGCGACTCAGATC-3'
	Reverse	5'-GGGAGGCTGCTGGTGAATATT-3'
	Probe FAM	5'-AGCCCCTGTTTGCTCCTCCGATAACTG-3'
hF.IX	Forward	5'-ATGACTTCACTCGGGTTGTTGG-3'
	reverse	5'- AGCCTCCACAGAATGCATCAAC-3'
Titin	forward	5'-AAAACGAGCAGTGACGTGAGC-3'
	Reverse	5'-TTCAGTCATGCTGCTAGCGC-3'
	Probe VIC	5'-TGCACGGAAGCGTCTCGTCTCAGTC-3'
Albumin	Forward	5'- GTTGCTGTTATCTCTTGTGGGCTGT-3'
	Reverse	5'- ACTCATGGGAGCTGCVCGGTTC-3'
CD8	Forward	5'- ATCACTCTCATCTGCTACC-3'
	Reverse	5'- GCCTTCCTGTCTGACTAG-3'
GAPDH	Forward	5'- CATGGCCTTCCGTGTTCCTA-3'
	Reverse	5'- GCGGCACGTCAGATCCA-3'
